# Proteomic analysis of heparin amelioration of cardiac injury after CA-CPR in rats: an exploratory proteomic study

**DOI:** 10.3389/fmed.2025.1657698

**Published:** 2026-01-07

**Authors:** Fa Wang, Biyun Tian, Ningkang Li, Yang Gu, Xiaoqin Li, Xiaohong Zhou, Yan Li, Xin Liu, Yinghua Gu, Yun Wang, Qingshan Ye

**Affiliations:** 1 Ningxia Medical University, Yinchuan, China; 2 Department of Anesthesiology, People’s Hospital of Ningxia Hui Autonomous Region, Ningxia Medical University, Yinchuan, China

**Keywords:** cardiac arrest, cardiopulmonary resuscitation, heparin, proteomic, autophagy

## Abstract

**Background:**

Patients who recover from cardiac arrest with autonomous circulation often have a poor prognosis due to myocardial dysfunction. Heparin, a pleiotropic drug, has not yet been identified for its myocardial protection mechanism in addition to anticoagulation. The aim of this study was to investigate the potential molecular mechanism by which heparin ameliorates cardiac injury after CA-CPR in rats via proteomic techniques.

**Methods:**

A rat asphyxiated CA-CPR model was established, and the rats were randomly divided into a sham operation group (Sham), a CA-CPR + normal saline group (NS) and a CA-CPR + heparin (H) intervention group. Myocardial histopathological changes were observed via HE staining, serum CK-MB levels were detected to assess the degree of myocardial injury, ultrastructural changes in cardiomyocytes were observed via transmission electron microscopy, and autophagy-related protein expression levels were detected via Western blotting. Data-independent acquisition (DIA) proteomics technology was used to analyze the differentially expressed proteins (DEPs) in the myocardial tissues of the three groups of rats, and GO functional annotation and KEGG pathway analysis were performed on the DEPs. Finally, Western blotting was used to verify the differential protein expression associated with mitochondria and autophagy.

**Results:**

Compared with those of the rats in group Sham, the myocardial tissues of the rats in group NS presented obvious pathological damage, with significantly elevated serum CK-MB and cTnI levels, severe damage to the ultrastructures of the cardiomyocytes, and decreased expression of autophagy-related proteins. Heparin intervention significantly improved these pathological changes and reversed the changes in the expression of some autophagy-related proteins. Proteomic analysis identified 6,002 proteins in total. When comparing NS vs. Sham groups, 198 proteins were differentially expressed (120 upregulated, 78 downregulated), while HP vs. NS comparison revealed 141 differentially expressed proteins (48 upregulated, 93 downregulated). Among these, 23 proteins showed significant expression changes both after CA-CPR and after heparin intervention. GO functional annotation and KEGG pathway analysis suggested that 5 proteins were potentially elated to autophagy. Western blot validation indicated that heparin intervention modulated the protein expression of IDH3A and RPTOR.

**Conclusion:**

This exploratory proteomic study suggests via proteomics the changes in myocardial protein expression network in heparin-attenuated cardiac injury in CA-CPR rats. This exploratory proteomic study provides preliminary evidence for heparin treatment of myocardial injury after CA-CPR. These findings are hypothesis-generating and require validation in larger cohorts and multi-time-point follow-up studies.

## 1 Introduction

Cardiac arrest (CA) is one of the leading causes of death and disability worldwide (1). Although advances in cardiopulmonary resuscitation (CPR) techniques have significantly improved the success rate of return of spontaneous circulation (ROSC), many CA patients still die after ROSC due to myocardial dysfunction (2). The pathological mechanisms of postresuscitation myocardial injury are complex and involve multiple factors, such as ischemia–reperfusion-induced mitochondrial dysfunction, oxidative stress, calcium overload, and imbalance in cellular autophagy (3). Currently, hypothermia is the only widely recognized postresuscitation myocardial protection strategy in the clinic, but its efficacy is limited by a strict time window and the potential risk of complications (4). Therefore, exploring safer and more effective interventions for myocardial protection is urgently needed in the field of resuscitation medicine.

Heparin, a classical anticoagulant, has long been used to prevent thrombosis during CPR (5). However, in recent years, an increasing number of studies have shown that heparin has pleiotropic functions beyond anticoagulation, including inhibition of complement activation, modulation of the inflammatory response, protection of endothelial cells, and modulation of cellular autophagy (6, 7). Our previous experimental study revealed that heparin pretreatment significantly ameliorated myocardial injury, improved cardiac function, and reversed mitochondrial damage in CA-CPR rats and that these protective effects may not be entirely dependent on its anticoagulant activity. In addition, retrospective clinical analyses suggest that heparin use during CPR in CA patients is associated with a better neurological prognosis (8). Nonetheless, current studies on the myocardial protective mechanism of heparin are mostly limited to a single signaling pathway or molecular target, and the lack of systematic molecular network analysis severely limits the depth and breadth of its clinical translational application.

Proteomics technology provides a powerful tool for systematically analyzing the molecular mechanisms of complex biological processes (9). Compared with traditional proteomics methods, data independence acquisition (DIA) significantly improves the accuracy and coverage of protein quantification via the cyclical acquisition of mass spectrometry signals from all peptides, and the core advantage of DIA lies in its “panoramic” data acquisition capability, which enables high-throughput protein identification and accurate quantification in a single experiment. The core advantage of DIA is its “panoramic” data acquisition capability, which enables high-throughput protein identification and accurate quantification in a single experiment and is especially suitable for the study of dynamic changes in complex biological samples. In addition, the data generated by DIA are highly traceable, which supports subsequent target validation and in-depth mining, providing a reliable data basis for mechanistic research (10, 11).

The significance of this study lies in the fact that through DIA proteomics technology, we were able to comprehensively analyze the protein expression changes in myocardial tissues after heparin intervention and reveal its key molecular mechanism to ameliorate myocardial injury after CA-CPR. This study provides not only a scientific basis for the nonanticoagulant clinical application of heparin but also new ideas for the study of other pleiotropic drugs and provides more effective options for clinical treatment.

## 2 Materials and methods

### 2.1 Animals

Adult male Sprague–Dawley (SD) rats (weighing 250–300 g, aged 8–12 weeks) were purchased from the Experimental Animal Centre of Ningxia Medical University. All experimental operations were approved by the Animal Ethics Committee of Ningxia Medical University (protocol code: IACUC-NYLAC-2023-237 and IACUC-H2025033), and the Guidelines for the Welfare and Use of Laboratory Animals were strictly followed. The rats were kept in a constant temperature (25 ± 2 °C) and humidity (50 ± 10%) environment with a 12-h light/dark cycle and free access to food and water.

### 2.2 Asphyxiated CA-CPR model establishment

We employed a well-established rat model of asphyxiated CA-CPR, the detailed surgical procedures and success criteria of which have been previously published (12). The general process is as follows.

#### 2.2.1 Anesthesia and intubation

After the rats were anesthetized with 5% isoflurane, tracheal intubation was performed via a visual small animal tracheostomy. The left femoral artery and femoral vein were cannulated via puncture, the mean arterial pressure was detected via arterial cannulation, and intravenous cannulation was used for backup drug administration. Electrocardiograms were monitored. The muscle relaxant cisatracurium besilate (0.15–0.2 mg/100 g) was intravenously administered while the small animal ventilator (tidal volume 1.5–2 mL/100 g, frequency 60–80 times/min) was connected.

#### 2.2.2 Asphyxia induction CA

Cardiac arrest was induced by clamping the airway tube and switching off the ventilator for 8 min. Cardiac arrest was confirmed by ECG and the loss of arterial pulses.

#### 2.2.3 Cardiopulmonary resuscitation

Chest compressions were performed at a frequency of 200 beats/min while ventilation was restored (100% oxygen, tidal volume of 1.5–2 mL/100 g, frequency of 60–80 beats/min). The restoration of sinus rhythm and the maintenance of a mean arterial pressure greater than 60 mmHg for more than 10 min are considered successful CPRs. Rats that did not achieve ROSC within 5 min of CPR were excluded from the study.

#### 2.2.4 Rats were randomly divided into three groups

Rats were randomly assigned to three groups using a computer-generated randomization schedule. The investigator performing the surgical procedures was blinded to group allocation during the experiment and outcome assessment. A total of 9 rats were initially enrolled in the study, of which 9 achieved successful ROSC and were included in the final proteomic analysis (Sham: *n* = 3, NS: *n* = 3, HP: *n* = 3).

Sham operation group (Sham group): only anesthesia intubation, puncture tube placement and other operations without asphyxia and CPR.

The CA-CPR + normal saline group (NS group) received CPR via immediate intravenous injection of the same volume of saline as heparin in the H group.

The CA-CPR + heparin intervention group (group HP) received intravenous heparin (6.25 U/100 g) immediately after CPR.

The sample size of *n* = 3 per group was chosen based on the established effect sizes observed in our preliminary studies and is common for initial, exploratory proteomic investigations. However, we acknowledge that this limits the statistical power and generalizability of the findings.

Heparin sodium injection (Sinopharm Rongsheng Pharmaceutical Co., Ltd., China, H20033326) was administered at a dose of 6.25 U/100 g (0.5 mg/kg) body weight. This dose was selected based on preliminary dose-ranging studies in our laboratory and literature reports demonstrating its efficacy and non-bleeding risk in the models (13, 14). While this dose is expected to achieve systemic anticoagulation, the primary focus of this study was to investigate its non-anticoagulant, pleiotropic effects. No overt bleeding complications were observed.

### 2.3 HE staining

Two hours after ROSC in the rats, the hearts were excised and fixed in 4% paraformaldehyde for 24 h. Sections (4 μm) were made after paraffin embedding and stained with hematoxylin–eosin (HE). Cardiomyocyte arrangement, necrosis and inflammatory infiltration were observed under a light microscope. Three samples were included in each group.

### 2.4 Serum CK-MB and cTnI measurement

Two hours after ROSC, 1 mL of whole blood was collected from each group of rats, which was stored at 4 °C overnight and then centrifuged at 3,000 rpm for 15 min at 4 °C. The supernatant was then collected and the CK-MB assayed by an automated biochemical analyzer (Chemray-800). Serum cTnI concentrations were measured using a specific rat ELISA kit (H149-2-1, Nanjing Jiancheng Bioengineering Institute, China). Briefly, serum samples and standards were added to a 96-well plate, followed by incubation with a detection antibody and a chromogenic substrate. The reaction was stopped, and the absorbance was read at 450 nm. Sample concentrations were interpolated from a standard curve. Each group contained three blood samples.

### 2.5 Transmission electron microscopy

Two hours after ROSC in the rats, the myocardial tissue was quickly removed, trimmed to 1 × 3 mm, and fixed in a 4% paraformaldehyde mixture for 24 h and 1% osmium acid for 2 h. Ethanol gradient dehydration, epoxy resin embedding, and ultrathin sectioning (70 nm) were performed. Mitochondrial swelling, crista fracture and autophagosome formation were observed via transmission electron microscopy (TEM). Three samples were included in each group.

### 2.6 Western blotting

Rat myocardial tissue was collected 2 h after ROSC, ground in liquid nitrogen and added to RIPA lysis buffer (containing protease inhibitors and phosphorylated protease inhibitors). After centrifugation, the supernatant was collected, and the protein concentration was determined via the BCA method. Thirty micrograms of protein was subjected to 10% SDS–PAGE and transferred to a PVDF membrane (0.45 μm). A primary antibody Beclin-1 (1:1000, RM0019, Biodragon, China) was used, and the samples were incubated at 4 °C overnight. The secondary antibodies used were HRP-labeled goat anti-rabbit IgG (1:5000) and incubated at room temperature for 1 h. The bands were developed via ECL ultrasensitive chemiluminescent solution, and ImageJ software was used to quantify the gray values of the bands, which were normalized to that of vinculin as an internal reference. WB validation of key proteins of the autophagy pathway was performed according to the proteomics results, and the procedure was the same as that used for the autophagy-related protein assay. The primary antibodies RPTOR (1:1000, BD-PN0044, Biodragon, China) and IDH3A (1:2000, BD-PE2677, Biodragon, China) were used, which were standardized with Tubulin as internal references. Each set of experiments was repeated three times.

### 2.7 DIA proteomics assay

#### 2.7.1 Total protein extraction

Myocardial tissues were taken from the rats in the sham operation group, CA-CPR with saline group and heparin intervention group (n = 3 in each group, taken 2 h after ROSC). Cardiac tissues were ground in liquid nitrogen, added to lysis solution (4% SDS, 100 mM Tris–HCl, pH 7.6, containing 1% DTT), and broken by ultrasonication in an ice bath (5 min, 5 s on/15 s off cycle). After denaturation at 95 °C for 10 min, 20 mM iodoacetamide (IAM) was added and alkylated away from light for 1 h. The proteins were purified via cold acetone precipitation (−20 °C for 2 h, −20 °C 12,000 rpm at 4 °C). The protein was purified by cold acetone precipitation (−20 °C precipitation for 2 h, centrifugation at 12,000 rpm for 15 min at 4 °C), and the precipitate was washed with cold acetone, dried under vacuum, and dissolved in 8 M urea buffer (100 mM TEAB, pH 8.5).

#### 2.7.2 Protein quality testing

The protein concentration was determined via the Bradford method (BSA standard curve of 0–0.5 μg/μL) in triplicate. Twenty micrograms of protein was subjected to 12% SDS–PAGE (80 V for 20 min for the concentrated gel and 120 V for 90 min for the separated gel), and protein integrity was verified via Coomassie Brilliant Blue staining.

#### 2.7.3 Proteolysis and peptide purification

Protein samples were reduced by dithiothreitol (DTT), trypsin (Promega) was added at 1:50 (w/w), digestion was carried out at 37 °C for 4 h, and then an equal amount of trypsin was added to continue the digestion for 16 h. Acidification was used to terminate the reaction (final concentration of 1% formic acid), and desalting was carried out on a C18 column (equilibrium of 0.1% formic acid, eluted with 70% acetonitrile), followed by lyophilization and storage.

#### 2.7.4 DIA mass spectrometric detection

System: Vanquish Neo UHPLC.

Column: PepMap™ Neo C18 (150 μm × 15 cm, 2 μm).

The mobile phases were as follows: Liquid A (0.1% formic acid aqueous solution) and Liquid B (80% acetonitrile/0.1% formic acid).

Gradient: 0–90 min, linearly increasing phase B to 35.

Instrument: Orbitrap instrument.

The ion source used was an easy-spray (ESI voltage of 2.0 kV, transfer tube temperature of 290 °C).

Scan mode: DIA full scan m/z 380--980 (resolution 240,000, AGC 500%).

The window settings were as follows: 300 isolation windows (2 Th/window) and 25% collision energy.

The secondary scan parameters were as follows: m/z 150–2000 (resolution 80,000, maximum injection time 3 ms).

### 2.8 Analysis of proteomics data

#### 2.8.1 Protein identification and quantification

The raw files were analyzed via DIA-NN software. The parameters were set as follows: precursor ions with a mass tolerance of 10 ppm and fragment ions with a mass tolerance of 0.02 Da. The immobilized modification was the alkylation modification of cysteine, and the variable modifications were the acetylation, the loss of methionine, and its acetylation derivatives, and a maximum of 2 missed cleavage sites were allowed.

To improve the quality of the analytical results, the results were further filtered by the DIA-NN. Peptide-spectrum matches (PSMs) with >99% confidence were retained, and only plausible peptide spectra and proteins were retained. Peptide and protein confidence was verified via an FDR ≤ 1% threshold. Differentially expressed proteins (DEPs) were defined as those with a |fold change| ≥ 1.2 and a Benjamini-Hochberg false discovery rate (FDR) adjusted *p*-value < 0.05.

#### 2.8.2 Functional analyses of proteins and DEPs

GO and IPR functional annotations (including Pfam, PRINTS, ProDom, SMART, ProSite, and PANTHER databases) were performed via interproscan software, and functional protein family and pathway analyses were performed on the identified proteins via COG and KEGG. Volcano plot analysis, cluster heatmap analysis and pathway enrichment analysis for GO, IPR and KEGG were performed for DEPs, and possible protein–protein interactions were predicted via STRING DB software.^1^

### 2.9 Statistical analysis

Statistical analysis was performed with GraphPad Prism 9.5. Data are expressed as the means ± standard deviations. For comparisons among three groups, one-way ANOVA followed by Tukey’s *post hoc* test was used. A *p*-value of less than 0.05 was considered statistically significant.

## 3 Results

### 3.1 Heparin improves myocardial structure and function after CA-CPR

**Figure 1:**
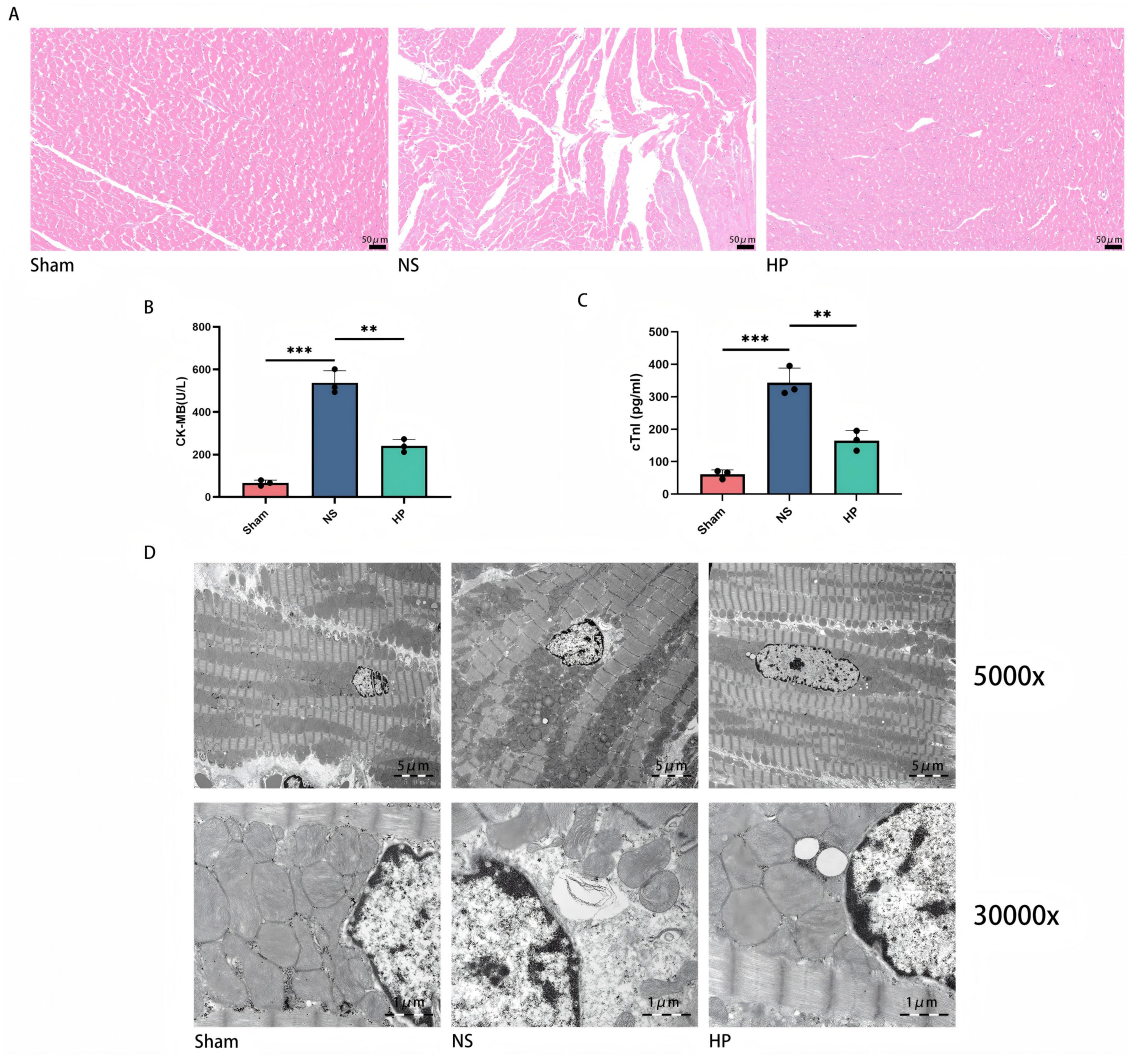
Effect of heparin on cardiac structure and function in CA-CPR rats. **(A)** Myocardial HE staining (200×). *N* = 3. **(B,C)** Serum levels of CKMB and cTnI. *N* = 3. The data represent the mean ± SD (**p < 0.01, ***p < 0.001). **(D)** TEM of myocardium (5,000 × and 30,000×). All data are from *N* = 3 biologically independent animals. Bar plots show mean ± SD with individual data points overlaid.

Myocardial histopathological changes were observed via HE staining. Myocardial structure was found to be normal in the Sham group. The myocardial cells in the NS group were disorganized, with significant interstitial edema and the formation of many necrotic vacuoles. In contrast, myocardial structural integrity was significantly improved, and necrotic areas were reduced in the HP group (Figure 1A). The results of the serum CK-MB and cTnI level assay revealed that the serum CK-MB and cTnI level was elevated in the NS group compared with the Sham group (*p* < 0.01), whereas the serum CK-MB and cTnI level was significantly reduced after heparin intervention (*p* < 0.01), suggesting that heparin effectively alleviated myocardial injury (Figures 1B,C). Transmission electron microscopy further revealed ultrastructural changes in cardiomyocytes, and the ultrastructure of the myocardium was approximately normal in the Sham group. Mofibrillar nodule fracture was obvious in the NS group, the mitochondria were obviously swollen, and the cristae were broken. In contrast, mitochondrial morphology was restored, and autophagic vesicles were found in the HP group, suggesting that heparin may attenuate myocardial injury by protecting mitochondrial function and modulating autophagy (Figure 1D).

### 3.2 Heparin regulates autophagy-related protein expression

**Figure 2:**
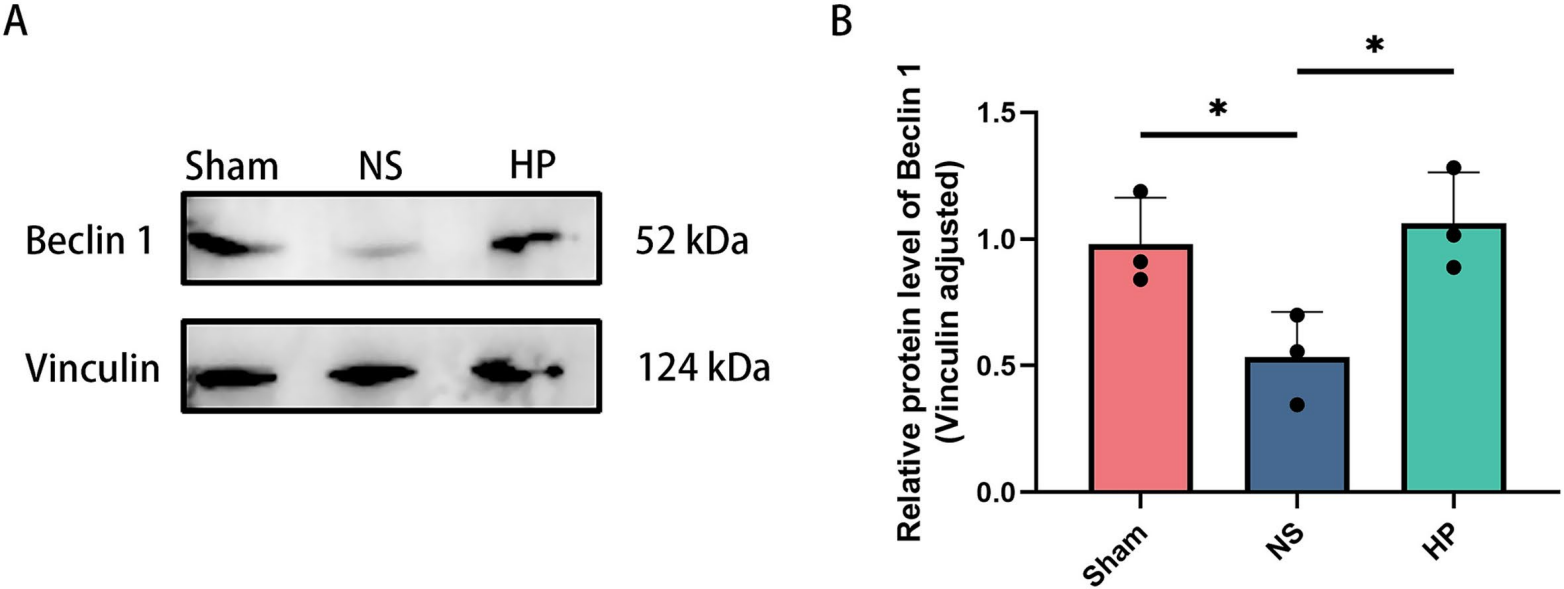
Detection of autophagy-related protein expression by western blotting. **(A,B)** Western blotting showing the expressions of Beclin 1. Data are from *N* = 3 biologically independent animals. Bar plots show mean ± SD with individual data points overlaid (*p < 0.05).

Western blot results revealed that Beclin1 expression was lower in the NS group than in the Sham group (*p* < 0.01). Beclin1 expression increased after heparin intervention (*p* < 0.01), suggesting that heparin may ameliorate myocardial injury by enhancing autophagic flux (Figures 2A,B).

### 3.3 Quality control of the proteomics data

**Figure 3:**
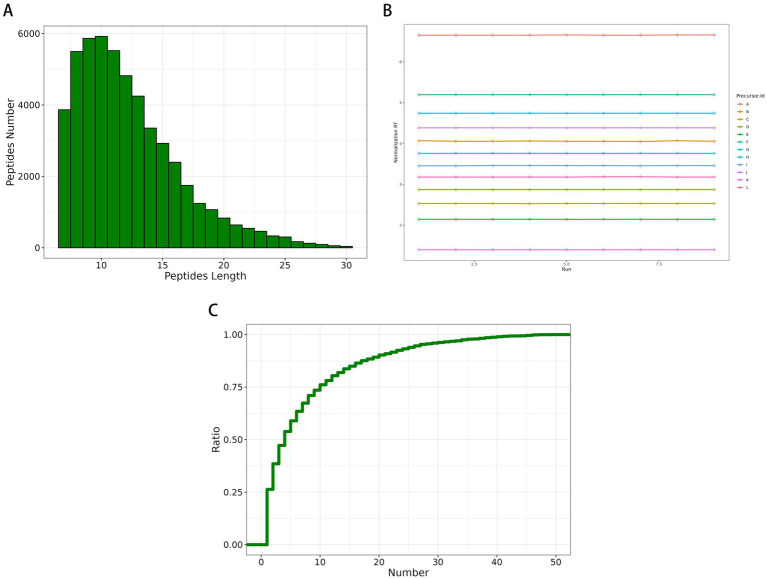
Quality control of data. **(A)** The distributions of peptide length. **(B)** iRT values of internal standard-corrected peptides. **(C)** Unique peptide number.

**Figure 4:**
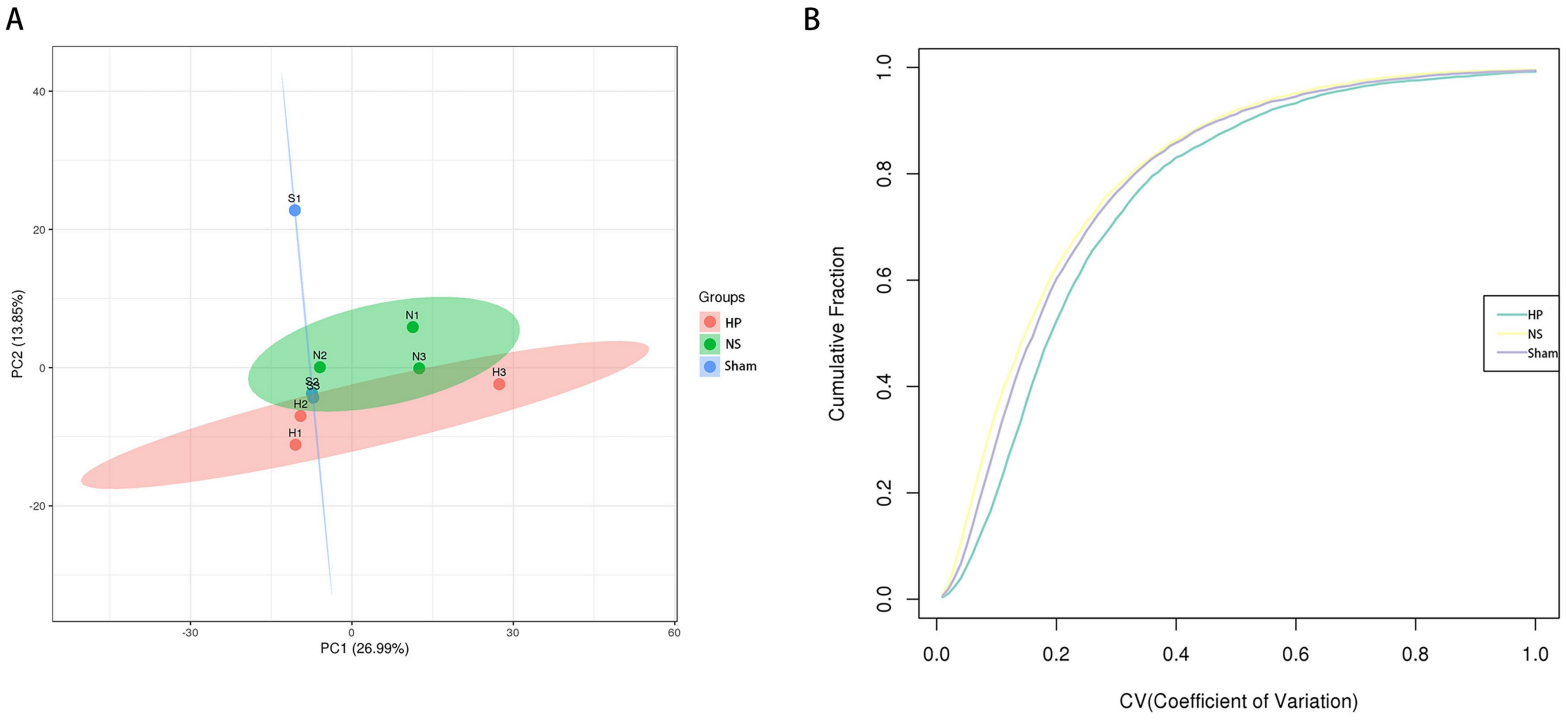
Quality control data for quantitative protein analysis. **(A)** Reproducibility of quantification measured using principal component analysis. **(B)** Reproducibility of quantification measured by coefficient of variation.

Quality control (QC) of data is a step toward obtaining results with stability and accuracy. For mass spectrometry data QC, data reproducibility and accuracy are assessed. The QC evaluation included peptide length distribution, iRT (Indexed Retention Time), and Unique peptide number distribution. A total of 52,096 peptides were identified in this study. The results revealed that most of the identified peptides were in the range of 7–20, indicating that the protease selected in this study was suitable (Figure 3A). The iRT represents a standardized retention time index (indexed retention time), and the iRT values of the internal standard-corrected peptides in this study were stable across the samples, suggesting that the data were well stabilized (Figure 3B). The distribution plot of the number of unique peptides revealed that the cumulative ratio of proteins containing unique peptides to total proteins increased slowly with increasing number of peptides, indicating that reliably enriched proteins were identified in this study (Figure 3C). The PCA results revealed that the overall protein differences between samples of each group and the variability between samples within a group were small (Figure 4A). Cumulative plots of the CV values for all proteins in each group of samples revealed a fast rising curve, suggesting good reproducibility of the samples as a whole (Figure 4B).

### 3.4 Identification of DEPs associated with CA-CPR and heparin administration

**Figure 5:**
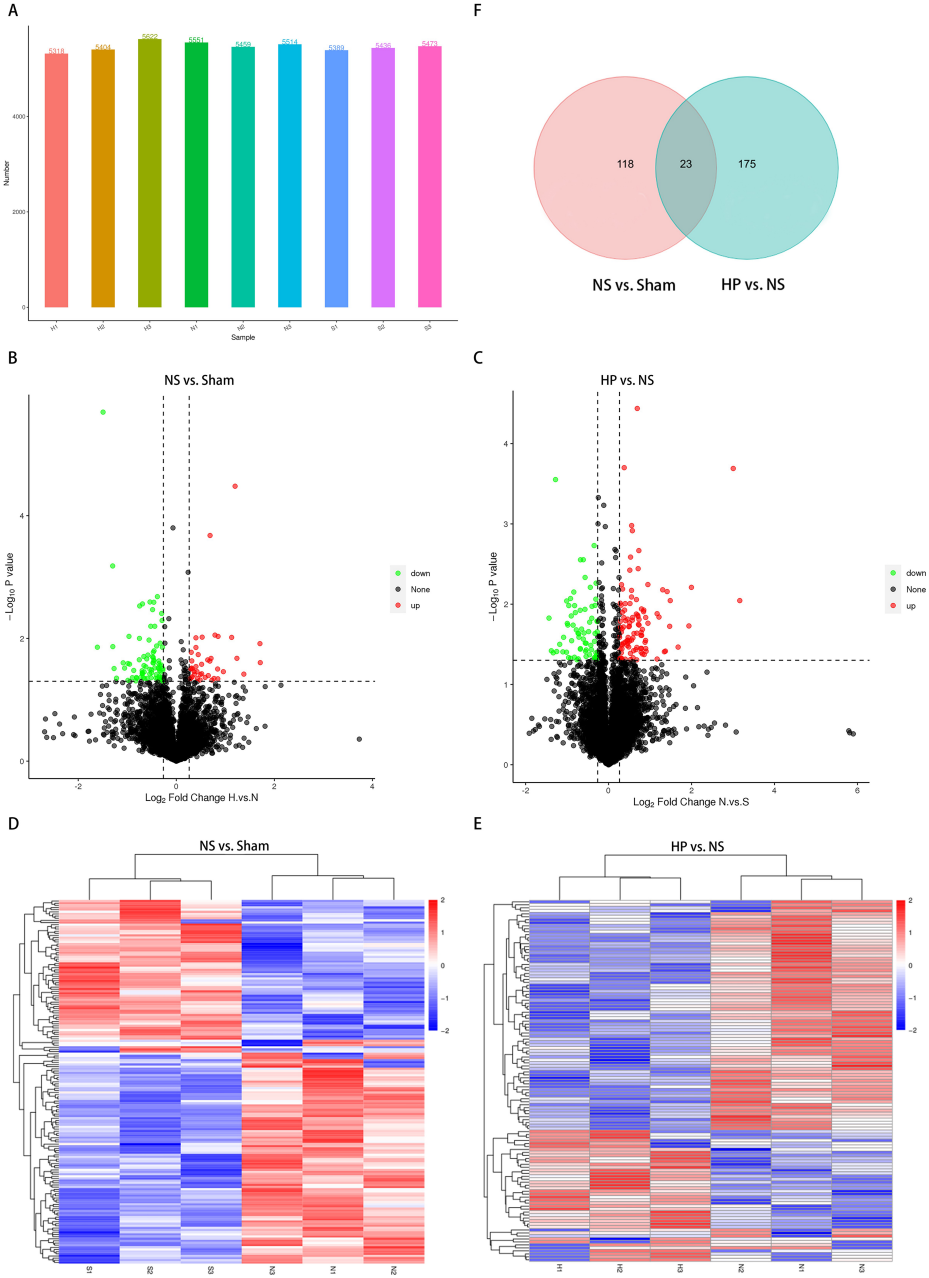
Results of the differentially-expressed proteins (DEPs) identified in the myocardial tissues. **(A)** Overview of sample protein identification. **(B)** The volcano plot illustrating the up-regulation (red) and down-regulation (blue) between the NS and the Sham group. **(C)** The volcano plot illustrating the up-regulation (red) and down-regulation (blue) between the HP and the NS group. **(D)** The heatmap showing the DEPs between the NS group and the Sham group. **(E)** The heatmap showing the DEPs between the HP group and the NS group. **(F)** The Venn diagram analysis showing the DEPs between NS verse Sham groups and HP verse NS groups.

This study revealed that the number of proteins identified by DIA was 6,002, and the number of proteins in each sample is shown in Figure 5A. In this study, differentially expressed proteins were defined as those with fold change > 1.2 (log2FC > 0.26) and a Benjamini-Hochberg FDR-adjusted *p*-value < 0.05 for upregulated proteins, and fold change < 0.83 (log2FC < −0.26) and a Benjamini-Hochberg FDR-adjusted *p*-value < 0.05 for downregulated proteins. On the basis of these criteria, when the NS group was compared with the Sham group, 198 differential proteins were obtained, including 120 upregulated proteins and 78 downregulated proteins (Figures 5B,D). A comparison of the HP and NS groups revealed 141 differential proteins, 48 of which were upregulated proteins and 93 of which were downregulated proteins (Figures 5C,E). We subsequently took the intersection of the differentially expressed proteins obtained from these two comparison groups and obtained 23 duplicate proteins (Figure 5F). That is, we wanted to determine which proteins changed both after CA-CPR and after immediate intervention with heparin during CPR. The 23 overlapping proteins, which were significantly altered in both NS vs. Sham and HP vs. NS comparisons (They are BLMH, CBFB, DDX46, ELAC2, ENPP3, ERLIN1, FABP6, FRA10AC1, IDH3A, MAT2B, MRC1, NIPSNAP1, NUP214, NUP35, NXF1, PAK2, PARD3B, RGD1306614, RPTOR, S100G, STBD1, TIPRL, ZFR). Detailed information on the differences of these genes and all data can be found via ProteomeXchange with identifier PXD071327.

### 3.5 Functional classification of differentially expressed proteins

**Figure 6:**
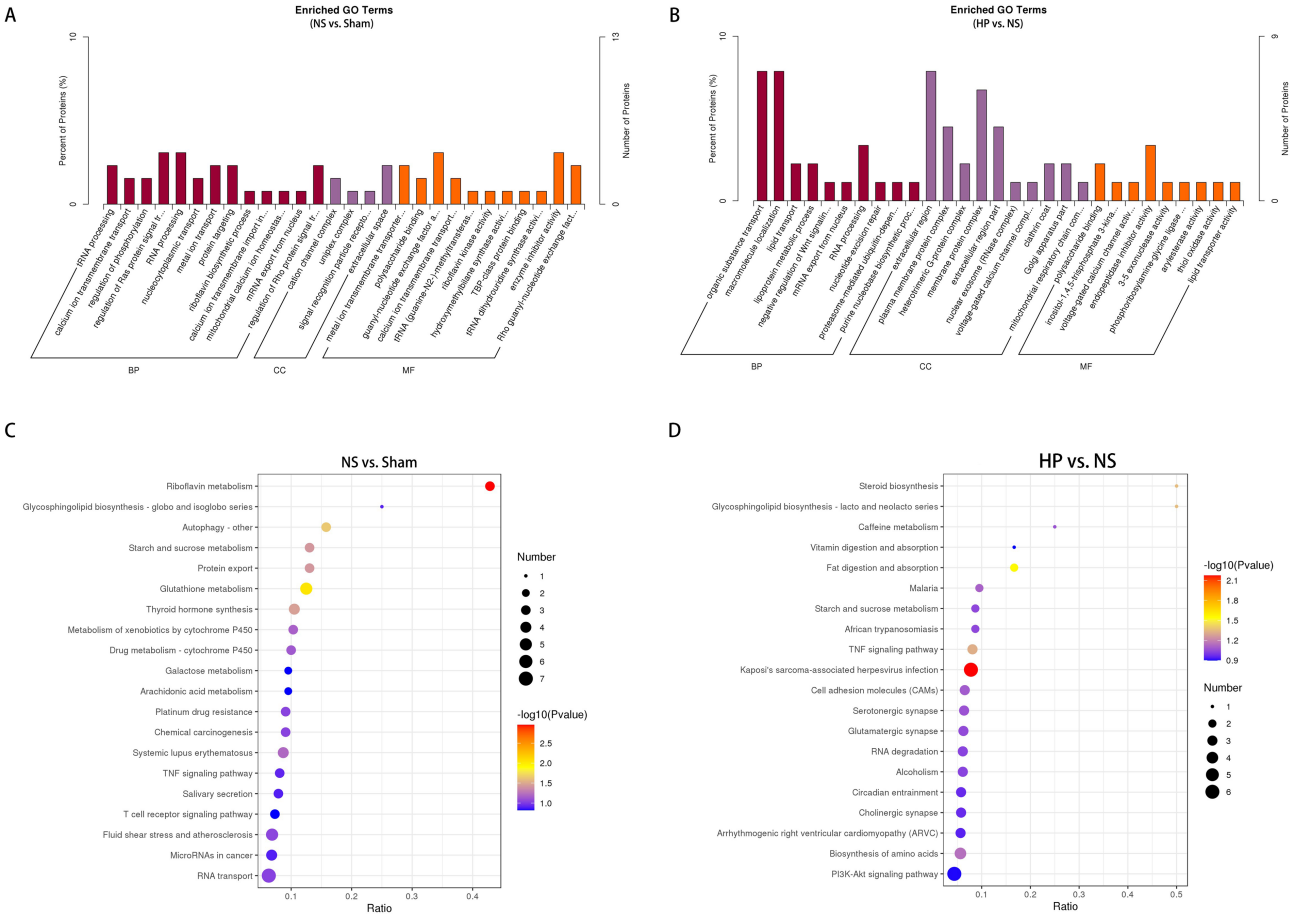
Bioinformatics analysis of DEPs. **(A)** Gene ontology enrichment analysis of DEPs between the NS group and the Sham group. **(B)** Gene ontology enrichment analysis of DEPs between the HP group and the NS group. **(C)** KEGG enrichment analysis of DEPs between the NS group and the Sham group. **(D)** KEGG enrichment analysis of DEPs between the HP group and the NS group.

To determine the potential effects of heparin treatment on DEPs and their cellular components and molecular functions in the hearts of CA-CPR rats, we performed GO enrichment analysis and KEGG enrichment analysis. GO enrichment included biological process (BP), cellular component (CC), and molecular function (MF) terms. GO analysis of the N group compared with the S group revealed that the DEPs associated with the regulation of Ras protein signal transduction, the extracellular space, and guanyl-nucleotide exchange factor activity were significantly associated with the DEPs (Figure 6A). KEGG analysis revealed that the major pathways associated with the DEPs included riboflavin metabolism, glutathione metabolism, and autophagy (Figure 6C). GO enrichment in the HP group compared with the NS group suggested that the DEPs were significantly associated with organic substance transport, the extracellular region, and endopeptidase inhibitor activity (Figure 6B). KEGG analysis revealed that the pathways associated with the DEPs included Kaposi’s sarcoma-associated herpesvirus infection, fat digestion and absorption, and the TNF signaling pathway (Figure 6D). Particularly, our analysis are focused specifically on the significantly enriched terms and pathways.

### 3.6 Analysis of the subcellular localization and structural domain enrichment of the differentially expressed proteins

**Figure 7:**
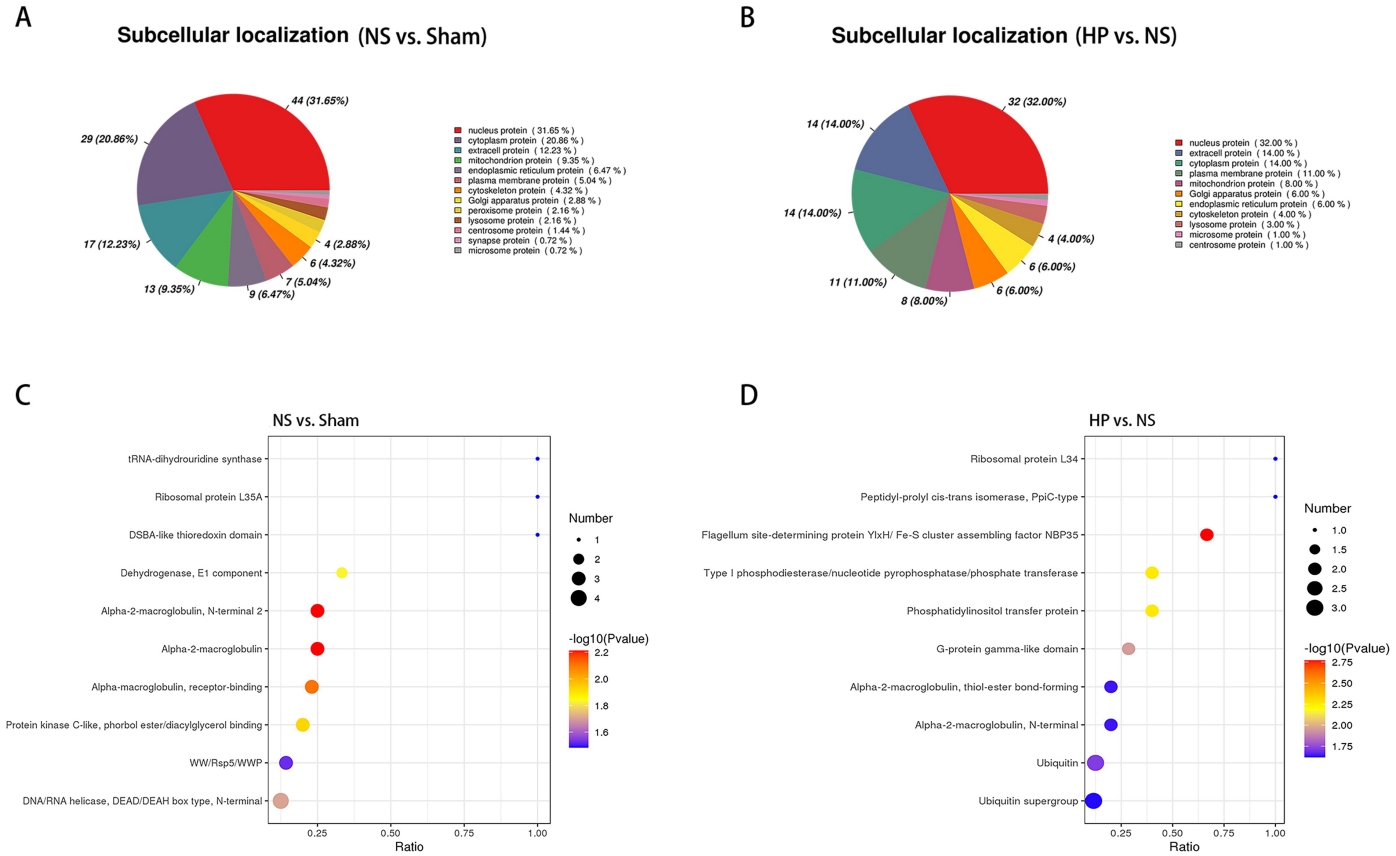
Subcellular localization and structural domain enrichment of DEPs. **(A)** Subcellular localization analysis of DEPs between the NS group and the Sham group. **(B)** Subcellular localization analysis of DEPs between the HP group and the NS group. **(C)** Structural domain enrichment analysis of DEPs between the NS group and the Sham group. **(D)** Structural domain enrichment analysis of DEPs between the HP group and the NS group.

In addition to functional changes, we focused on the subcellular localization of DEPs in CA-CPR rats and the structural domain enrichment of DEPs after heparin treatment. Compared with those in the Sham group, the DEPs in the NS group were located mainly in nuclear proteins (31.65%), cytoplasmic proteins (20.86%) and extracellular proteins (12.23%) (Figure 7A). The structural domain enrichment was mainly concentrated in alpha-2-macroglobulin, alpha-2-macroglobulin, and the N-terminal 2 (Figure 7C).

Compared with those in the NS group, the DEPs in the HP group were located mainly in nuclear proteins (32.00%), extracellular proteins (14.00%) and cytoplasmic proteins (14.00%) (Figure 7B). Structural domain enrichment was mainly concentrated in the flagella site-determining protein YlxH/Fe-S cluster assembly factor NBP35 and the phosphatidylinositol transfer protein (Figure 7D).

### 3.7 Protein interaction network analysis

**Figure 8:**
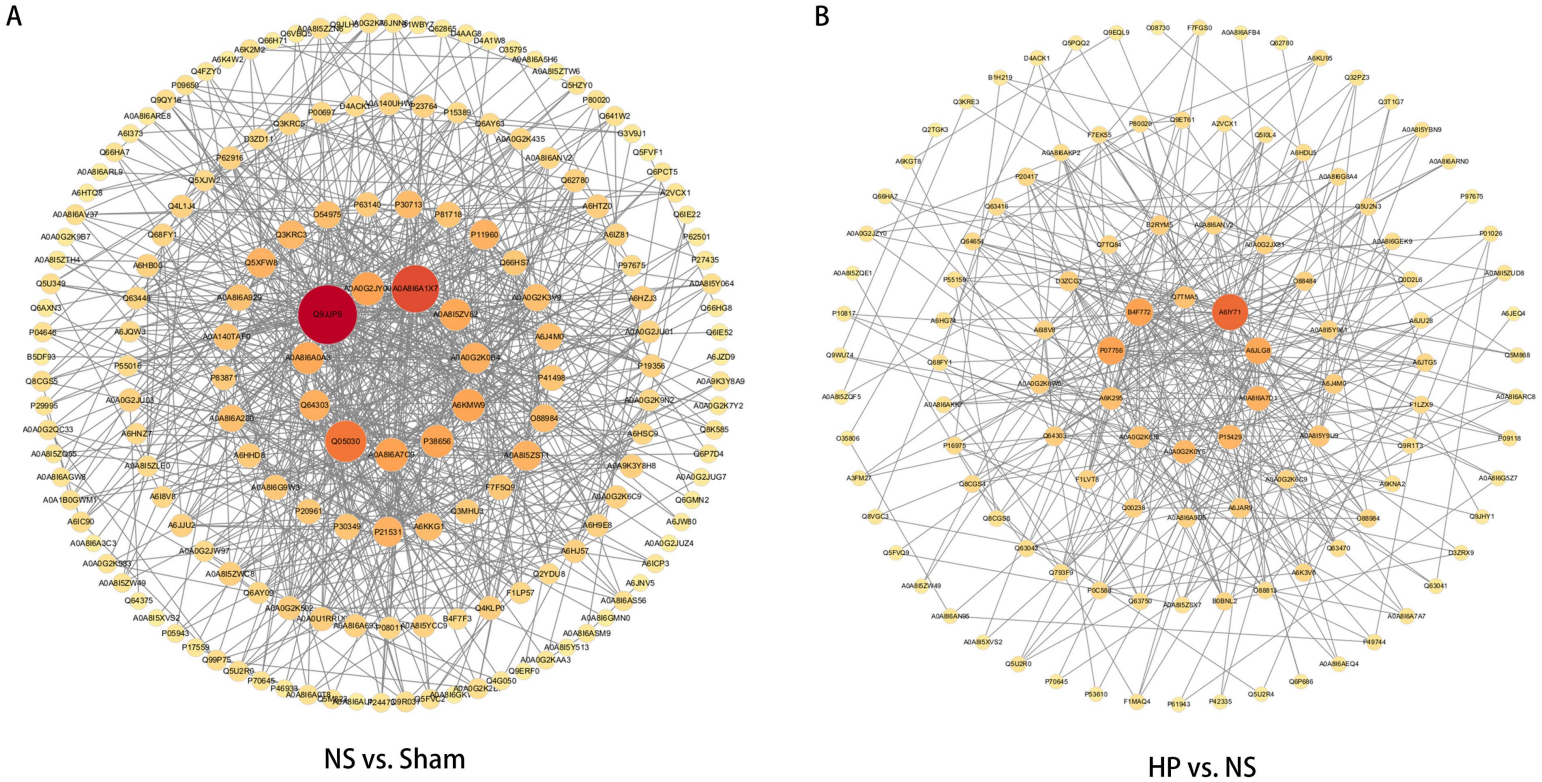
Protein–protein interaction networks of DEPs. **(A)** PPI network between the NS group and the Sham group. **(B)** PPI network between the HP group and the NS group.

Next, the StringDB protein interaction database^2^ was used to carry out interaction analysis of the identified proteins. If there was a corresponding species in the database, the sequences of the corresponding species were extracted directly; if not, the sequences of the near-origin species were extracted, and then the sequences of the differential proteins were compared with the extracted sequences in BLAST to derive the corresponding interaction information and construct network diagrams (Figures 8A,B). The proteins with the highest scores in the NS group compared with the Sham group were F1LP57 & A0A8I6A280 (gene name: MAP2K4 & MAP3K5, score: 979), P20961 & A6KKG1 (gene name: SERPINE1 & ALB, score: 975), and P04646 & P21531 (gene name: RPL35A & RPL3, score: 974). The highest scoring reciprocal proteins in group HP compared with those in group NS were A0A8I6ANV2 & Q0D2L6 (gene name: RPTOR & RRAGC, score: 997), Q8CGS4 & Q793F9 (gene name: CHMP3 & VPS4A, score: 994), and A6JU28 & A0A8I6A9D5 (gene name: EXOSC2 & EXOSC10, score: 980).

### 3.8 Validation of potential targets of myocardial injury in rats after heparin amelioration by CA-CPR

**Figure 9:**
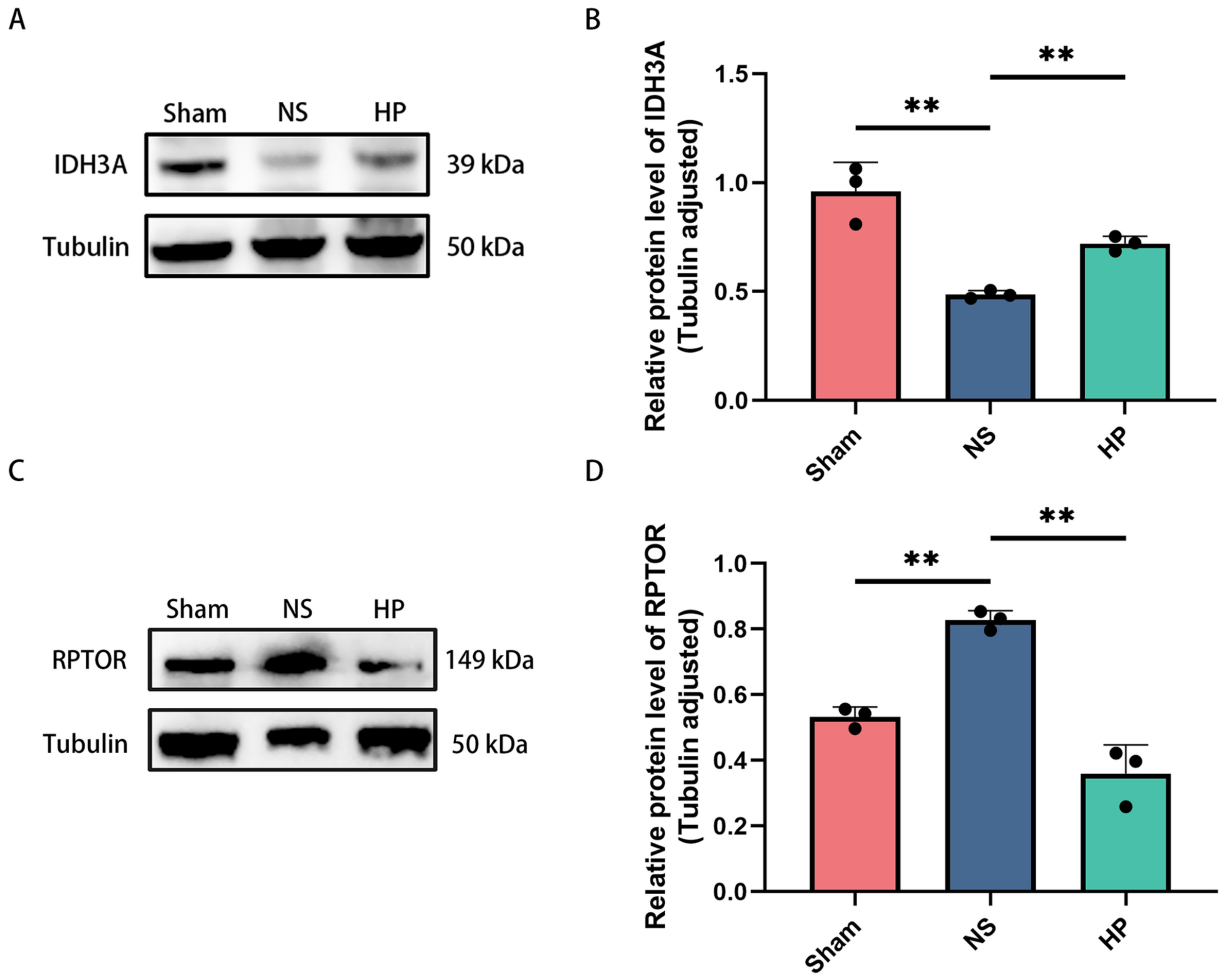
Validation of the overlapping proteins by western blotting. **(A,B)** Western blotting showing the expressions of IDH3A. *N* = 3. **(C,D)** Western blotting showing the expressions of RPTOR. Data are from *N* = 3 biologically independent animals. Bar plots show mean ± SD with individual data points overlaid (*p < 0.05, **p < 0.01).

Owing to the improved myocardial mitochondrial structure and changes in autophagy in CA-CPR rats after heparin intervention, we screened 1 protein with subcellular localization to mitochondria (IDH3A) and 4 proteins directly implicated in autophagy function (RPTOR, STBD1, TIRPL, NIPSNAP1) from the overlapping differential proteins. Characterizing these 5 proteins, we identified 2 significantly differentially expressed proteins, IDH3A and RPTOR. Compared with that in the Sham group, the expression of IDH3A was decreased, and the expression of RPTOR was increased in the NS group, while heparin administration reversed the changes in the expression of both proteins (Figures 9A–D).

## 4 Discussion

Myocardial injury triggered by the recovery of the autonomic circulation after cardiac arrest is a central pathological aspect of death and disability after resuscitation (15). The mechanism is complex and involves a vicious cycle of mitochondrial dysfunction, reactive oxygen species (ROS) bursts, calcium overload and autophagy imbalance (3). As the core of energy metabolism and apoptosis regulation, mitochondrial damage directly leads to ATP synthesis deficiency and cell death signaling activation (16). In recent years, therapeutic strategies targeting autophagy regulation and metabolic reprogramming have gradually become a research hotspot, but clinical translation still faces the challenge of insufficient target specificity (17). Heparin, a pleiotropic drug, has been reported for its nonanticoagulant mechanisms (e.g., anti-inflammatory, antioxidant) in ischemia–reperfusion injury (18), but its protein molecular network in myocardial injury after CA-CPR has not been systematically resolved. In the present study, we indicated that heparin may exerts cardioprotective effects through the regulation of metabolism and autophagy via DIA proteomics analysis and identified two key effector molecules, IDH3A and RPTOR.

In the present study, the cardioprotective effect of heparin was evaluated by establishing an asphyxiated CA-CPR rat model. HE staining revealed that heparin intervention significantly attenuated CA-CPR-induced cardiomyocyte necrosis and interstitial edema. Serum CK-MB and cTnI levels further confirmed that heparin caused a significant reduction in the expression of markers of myocardial injury, suggesting a clear pathological ameliorative effect of heparin. Transmission electron microscopy revealed clues to the mechanism at the subcellular level. The mitochondrial cristae were disrupted and the matrix vacuolated in the CA-CPR group, whereas the mitochondrial structure was largely restored, and autophagic vesicles were observed in the heparin group. These observations are consistent with a role for heparin in modulating mitochondrial homeostasis and autophagy-related processes, although autophagic flux was not directly assessed. Western blot analysis revealed that heparin intervention significantly upregulated the expression of Beclin1, suggesting that modulation of autophagy may be involved in the protective mechanism of heparin. However, comprehensive assessment of autophagic flux using additional markers such as LC3B-II/I and p62 would be needed to confirm this hypothesis.

We identified 6,002 proteins via proteomic analysis via DIA technology, of which 198 DEPs were present between the N and S groups, whereas 141 DEPs were present between the H and N groups. Taking the intersection of these two differentially expressed protein groups, another 23 proteins were obtained. These 23 DEPs were altered both in CA-CPR and at the same time after heparin intervention. GO and KEGG enrichment analyses revealed that these DEPs were enriched mainly in the tricarboxylic acid (TCA) cycle, autophagy regulation, and oxidative stress-responsive pathways. Of these 23DEPs, five were associated with mitochondria and autophagy. Finally identified by western blotting, we revealed two core proteins. Notably, isocitrate dehydrogenase 3α subunit (IDH3A) and rapamycin target protein-regulated related protein (RPTOR) were identified as core targets of heparin action.

IDH3A is the rate-limiting enzyme in the TCA cycle, catalyzing the oxidative decarboxylation of isocitric acid to generate α-ketoglutarate (α-KG), as well as the generation of NADH, which provides reducing power for the mitochondrial electron transport chain (19). In the present study, we found that heparin intervention significantly upregulated IDH3A expression, suggesting that heparin may promote ATP production and alleviate impaired myocardial energy metabolism after CA-CPR by increasing TCA cycle activity. Notably, α-KG not only is an energy metabolism intermediate but also serves as a cofactor for epigenetic modification and affects inflammation-related gene expression by regulating histone demethylase activity (20). This mechanism may partially explain the role of heparin in attenuating oxidative stress and inflammatory responses.

RPTOR is a core component of the mTORC1 complex and is involved in the regulation of cell growth, autophagy and metabolic adaptation (21, 22). The present study revealed that RPTOR expression was downregulated after heparin intervention, which may relieve its inhibitory effect on autophagy and promote autophagosome formation by inhibiting mTORC1 activity (consistent with Beclin 1 accumulation). In addition, the mTORC1 pathway is closely related to mitochondrial function, and its inhibition activated PGC-1α to increase mitochondrial biosynthesis and antioxidant capacity (23, 24). Combined with the restoration of mitochondrial morphology observed via transmission electron microscopy, heparin may ameliorate myocardial injury by coordinating autophagy and mitochondrial homeostasis via the RPTOR/mTORC1 axis.

Protein interaction network (PPI) analyses further revealed the multitarget nature of heparin action. STRING database predictions revealed that RPTOR with RRAGC (score: 997), CHMP3 with VPS4A (score: 994), and EXOSC2 with EXOSC10 (score: 980) were the three highest scoring groups of proteins after heparin intervention. RRAGC is the gene encoding Ras-related GTP-binding protein C, which belongs to the Rag GTPase family. As part of the Rag complex, RRAGC activates the mTORC1 signaling pathway by binding to mTORC1 (25). CHMP3 is a charged multivesicular body protein 3 that is involved mainly in intracellular membrane transport and protein sorting processes. VPS4A is a vesicular protein sorting 4 homolog A, which is mainly responsible for intracellular protein transport and cell membrane repair (26). EXOSC2 and EXOSC10 are exosome components 2 and 10, respectively, and EXOSC2 is involved mainly in RNA metabolism, cell growth and gene expression regulation, whereas EXOSC10 plays important roles in RNA metabolism, DNA repair and adaptive cellular responses (27).

A key consideration in interpreting our results is distinguishing heparin’s direct cellular effects from its anticoagulant actions. The dose used here is known to produce therapeutic anticoagulation. Therefore, while we cannot entirely rule out the contribution of improved microcirculatory flow due to anticoagulation, the proteomic identification of specific intracellular pathways (e.g., TCA cycle, mTORC1/autophagy) suggests that direct cardioprotective mechanisms are likely at play.

Although the present study preliminarily revealed the cardioprotective mechanism of heparin in CA-CPR rats, several limitations remain. First, the mechanisms regulating the expression of IDH3A and RPTOR have not been fully clarified, and whether heparin binds these proteins directly or regulates them indirectly through upstream signaling molecules (e.g., AMPK and SIRT1) still needs to be verified. Second, proteomics data reflect only mRNA–protein expression correlations and need to be combined with phosphoproteomics data to reveal dynamic changes in posttranslational modifications (e.g., the phosphorylation status of mTORC1). Third, the sample size (n = 3 per group), though common in exploratory proteomic studies, limits the statistical power and generalizability of our conclusions. Fourth, the single early time point (2 h post-ROSC) captures immediate responses but may not reflect longer-term molecular changes. In addition, while we focused on heparin’s non-anticoagulant effects, we cannot completely exclude contributions from its anticoagulant properties. Future studies with larger sample sizes, multiple time points, and direct comparison with other anticoagulants would help address these limitations. Furthermore, future studies could be conducted in depth in the following directions: (1) to validate the functional necessity of the AAV-mediated cardiac-specific knockout model of IDH3A/RPTOR; (2) to analyze the dynamic changes in metabolites, such as *α*-KG and the NADH/NAD + ratio, via metabolomics to clarify the spatial and temporal characteristics of metabolic reprogramming; (3) to explore the potential for synergistic therapies involving heparin and existing cardioprotective agents; and (4) to conduct a clinical cohort study to detect the correlation between serum IDH3A/RPTOR levels and heparin efficacy in CA-CPR patients.

In summary, in this study, the proteins in the myocardial tissues of CA-CPR rats after heparin intervention were comprehensively investigated via DIA proteomics technology. A total of 198 DEPs were detected in group NS compared with group Sham. A total of 141 DEPs were detected in group HP compared with group NS. Eventually, IDH3A and RPTOR were emerged as key proteins. These findings suggest that heparin may ameliorate cardiac injury in rats after CA-CPR by regulating metabolism and autophagy. These findings provide a new theoretical basis for the nonanticoagulant pleiotropic application of heparin and lay a molecular foundation for the development of precise therapeutic strategies for postresuscitation myocardial protection. However, the specific molecular mechanisms of these proteins during CA-CPR and their interactions with other proteins require further research. Future studies need to combine gene editing and metabolomics technologies to elucidate the molecular associations between heparin and its target proteins and functional phenotypes.

## Data Availability

Original datasets are available in the publicly accessible repository ProteomeXchange: The original contributions presented in the study are publicly available. This data can be found here: [http://www.ebi.ac.uk/pride] and [accession number: PXD071327].

## Footnotes

1
http://STRING.embl.de/

2
http://string-db.org/
